# Explaining migration intention from selected psycho-social variables in South Wollo, Ethiopia

**DOI:** 10.3389/fsoc.2022.960203

**Published:** 2022-12-05

**Authors:** Abebe Kibret Assfaw, Abebaw Minaye

**Affiliations:** ^1^Department of Psychology, Institution of Behavioral Science and Teachers' Education, Wollo University, Dessie, Ethiopia; ^2^School of Psychology, College of Education and Humanities, Addis Ababa University, Addis Ababa, Ethiopia

**Keywords:** attitude: migration intention, national pride, perceived behavioral control, place attachment, social network and subjective social norm

## Abstract

Migration has become one of the challenging global issues of the twenty-first century. Therefore, analyzing the intention to migrate is essential to develop clear strategies to manage actual migration. This research aimed to investigate emigration intention predictors (namely place attachment, attitude, national pride, subjective social norm, and perceived behavioral control) and the interaction effect of social networks between those predictors and migration intention. A sample of 701 high school students participated in the research. Exploratory Factor Analysis and Confirmatory Factor Analysis were employed to explore the dimension and loading of items respectively. To test the moderation effect of social networks on migration intention and the predictor variables, structural equation modeling was employed using Amos version 27. The findings indicated that Attitude, National pride, subjective social norm, and perceived behavioral control predict youths' emigration intention. The result also revealed that social network strengthens the negative correlation between place attachment and intention to migrate only if the individuals have a high social network. The social network also moderates the relationship between attitude and emigration intention.

## Introduction

Migration has become one of the challenging global issues of the twenty-first century. Though it is as old as human beings, research findings showed that its magnitude, complexion, and its aversive consequences have been increasing from time to time. It is stated that migration dynamics are continually evolving and new migration flows develop due to different push and pull factors like economic, social, and political structures in host and sending countries (Kuschminder et al., 2012). The high rate of migration has been observed in terms of intensity and extensiveness due to social, economic, and political reasons across the world (Gurieva et al., 2015). Similarly, O'Reilly (2012) indicated that international migration has been increasing in the last 30 years. It is also stated that in an era of globalization, migration is being experienced in all states and from people in all corners of the world (IOM., 2017). The number of international migrants across the world reached about 281 million in 2020 and could form the fourth most highly populated country if they came together (IOM., 2022). It was also indicated that the number of migrants globally is expected to reach over 400 million by 2050 (Martin, 2013).

One of the variables in studying migration is the intention of migrants to leave their countries. The extent to which it predicts actual migration has been one of the centers of research in the area. It is considered an important predictor of the realization of migration (Jong et al., 1986). Similarly, Simmons indicated that intention is a strong predictor of the future migration of individuals (Simmons, 1986). Another research finding by Böheim and Taylor (2002) pointed out that individuals who showed early migration intention are more likely to migrate than others who did not show intention to migrate. Approximately 710 million people around the world expressed their general desire to migrate and among the people who showed a general desire to migrate, 66 million of them indicated that they were preparing to migrate within 12 months (Laczko et al., 2017). Gordon and Molho (1997) also found out that a large proportion of people who developed the intention to migrate from the United Kingdom left their country within 5 years. Sandu and Jong (1998) also showed that intention is an essential determinant factor in the decision-making process of migration. In the most recent study conducted in more than 160 countries, the findings showed the presence of a strong association between migration intention and actual migration (Tjaden et al., 2019).

Previous research investigated different factors that contribute to the cross-border migration of individuals. The most frequently researched causes of migration are economic, social, and political issues. People most commonly migrate for two main reasons. First, people migrate to improve their economic status. Second, they migrate to get together with the family members who migrated previously (Massey et al., 1993). There have been research findings that indicate that migration does not only occur due to economic problems but there are social and psychological factors that put pressure on individuals to move from one place to another. Place attachment has multidimensions (place dependency, place identity, place effect, and place social bonding; Ramkissoon et al., 2012) and has been getting attention for its effect on attitude and behaviors. For example, place attachment plays an essential role in determining individuals' pro-social and pro-environmental behavioral intention (Ramkissoon, 2020b) and engagements in desirable behaviors (Ramkissoon, 2020a).

Factors like place attachment and having parents and friends in the place of birth also play roles in an individual's decision whether to migrate or to stay (Frieze et al., 2011). These researchers found that individuals who have a strong attachment to their home country or residence areas, together with having many friends, have an increased possibility of staying in those places or regions. On the other hand, individuals who lack strong place attachment and have few friends are highly susceptible to migration and migration intention. Individuals with lower place attachment have a positive attitude toward migration (Petrović et al., 2017). Other researchers tried to see the role of place attachment in settlement intention in other ways. For example, a study conducted in Poland showed that there is a positive relationship between place attachment and settlement intention. Individuals with high place attachment develop the intention to stay where they are (Toruńczyk-Ruiz and Brunarska, 2018). On the contrary, other research findings revealed that strong place attachment by itself is not enough to keep adolescents in their origin areas/places. It is indicated that even though adolescents between grades eight and eleven have strong place attachments, they are not willing to stay in those places. When adolescents are frustrated due to their perception of a lack of jobs, place attachment becomes less important to make them stay at their place of birth or where they grew up (Elder et al., 1996). It was also found that place attachment is not enough for adolescents to stay in the place they grew up (Eacott and Sonn, 2006). They found that individuals with strong place attachment have a propensity to leave the place they grew up if they failed or feel unable to achieve other needs they set as a priority. As a result, their research study indicated that place attachment is not an important factor that determines individuals' decision to migrate.

Another psychological variable that is important to consider in migration intention research is the concept of attitude toward migration. Attitude toward migration is an important driving force for migration. Attitude toward migration is the most important predicting factor of the intention to migrate which is justified as a means to improve the person's and their family's material standard of living (Minaye and Zeleke, 2017). Migration is perceived as means of improving the lives of family members by many young persons and as a result of this, they showed a strong propensity to migration (Ommundsen et al., 2010). A similar effect of attitude toward migration in determining intention was found in Russia. Attitude toward migration plays role in determining migration intention. Individuals with a positive attitude toward migration have a high degree of migration intention to other countries (Chuvashov, 2014). Social psychological factors such as attitude toward migration, subjective norms and anticipated constraints to migrate are essential determinants of migration intention. Individuals' perception that others need them to migrate has a significant correlation to their development of migration intention. In addition, their confidence in their ability to overcome the migration process easily increases their tendency of developing migration intention (McHugh, 1984). Silva and Neto also indicated how the attitude toward migration determines the intention to move. They found that individuals with a positive attitude toward migration develop a migration intention more than individuals who have a negative attitude toward migration (Silva and Neto, 1983).

There have been other research findings that indicated the role that psychological variables play in contributing to an individual's migration intention. Self-efficacy is an important predictor variable that explains the migration intention of individuals. Their finding indicated that individuals with high self-efficacy have a high degree of migration intention compared to individuals with low self-efficacy (Groenewold et al., 2012). Individuals' belief about their capacity to perform a given activity in a given situation determines their engagement to experience unfamiliar situations. This general explanation also works for individuals' migration intention. Individuals who have high self-efficacy believe that they can manage any problems associated with migration and have a stronger intention to migrate than individuals with low self-efficacy (Sherer et al., 1982).

Another psychological variable that determines migration intention is national pride. National pride is the positive feeling individuals have about their respective countries that are derived from one's national identity. It is both the sense of esteem a person has for his or her nation and the self-esteem he or she acquires from his or her national identity (Smith and Kim, 2006). Different researchers indicated that individuals with a strong national identity have a weak propensity to develop migration intention. The increasing level of national identity is negatively correlated with migration intention. The stronger the national identity of the individual, the weaker the migration intention (Chuvashov, 2014). National pride predicts the migration intention of individuals. Migration intention is negatively correlated to national pride indicating that as national pride increases, migration intention decreases (Chan-Hoong and Soon, 2011).

Youths' perceptions of subjective social norms and how significant others expect them to behave about migration play a role in migration intention. It determines individuals' emigration intention. It predicts the migration behavior of individuals through migration intention (Gödri and Feleky, 2017). The out-migration and migration intention of males and females is positively predicted by subjective social norms (DeJong, 2000). Components of the theory of planned behavior such as attitude, subjective social norms, and perceived behavioral control predict individuals' migration intention (Willekens, 2021). The unspoken belief of the community, whether real or perceived, impacted individuals' decisions either to migrate to other countries or stay in their home country. Young people perceive that if they stayed in their community, they will be viewed negatively by their society which in turn imposes the intention to migrate on adolescents (Eacott and Sonn, 2006).

Network Theory of Migration assumes that the cause of migration is not purely economic. It is a theory that explains migration in relation to the ties or connections between/among individuals in the home and destination countries. It is stated that migration is caused by the interpersonal ties which connect the potential migrants, the already migrated individuals, and non-migrants in the origin and destination countries through different linkages such as kinship, friendship, and shared community origin. A social network is increasingly regarded as an important source of social capital (Ryan et al., 2008) that minimizes the cost of migration, maximizes the returns the migrants could achieve from migration, and facilitate finding jobs in the destination countries (Massey et al., 1993). The mere existence of a network and migration chain reduces the risks and costs the migrants might experience which, in turn, increases migration. The previous data on migrants provide accurate information about the reality like the available employment opportunity in the destination countries for the potential migrants (Zaiceva and Zimmermann, 2009). Similarly, Van Dalen, Groenewold, and Schoorl stated that one's social network is influential in determining migration and migration intention in some countries like Ghana and Egypt. But it did not have any impact on influencing individuals to develop migration intentions in Morocco and Senegal (van Dalen et al., 2005). Most previous research findings indicate social networks are a predictor of migration and migration intention. Among psychological variables that predict migration and migration intention, attitude (Minaye and Zeleke, 2017), subjective social norm (Gödri and Feleky, 2017), social network, and perceived behavioral control (Groenewold et al., 2012) are some that previous researchers have examined. But having positive attitude toward migration, perceived behavioral control of individuals to migrate and subjective social norms could be enhanced when an individual has a high social network in the destination country. When individuals have a high social network with individuals who have previously migrated abroad, they might think that they would be given psychological support, temporal house accommodation, coverage of transportation, and assistance in finding jobs. These, in turn, strengthen their attitudes toward migration thereby increasing their perceived capability of migrating and enhancing their tendency to accept the perceived social pressure to migrate. In line with this, as the push-pull framework indicates, migration is deterred if individuals do not have friends and relatives in the destination country (Rainer and Siedler, 2009). Therefore, one's social network could interact with other psychosocial variables to influence individuals' migration intention and the consequent migration behavior.

Research findings indicate that the most dominant migrants in Ethiopia are the younger segment of the society. For example, Kuschminder et al. (2012) stated that the young and primary children of the family are the dominant migrants in Ethiopia. Young people from Ethiopia's vast rural areas are extensively recruited by their friends, relatives, and brokers with the promise of a better life and a good job abroad in general and in the Middle East in particular. Different research findings indicated that due to numerous reasons, there have been massive flows of regular or irregular migrants from Ethiopia to other countries. A considerable number of Ethiopian migrant workers migrated to Saudi Arabia in search of work through regular and irregular channels (Zemene, 2015) and it is roughly estimated that about 75,000–100,000 people migrate each year from Ethiopia into Sudan, Libya, and other nearby Arab countries (Aberra and Gebeyehu, 2011). Approximately 44% of Ethiopian migrants prefer the Middle East while 30% of them are directed to the West (North America, Europe, and Australia). It was found that the dominant migrants to the Middle East are females and less educated individuals. On the contrary, in general, males and more educated individuals, in particular, migrated to western countries. Moreover, migrants from Ethiopian urban areas preferred to migrate to the West, while migrants from the rural part of Ethiopia migrate to Africa and other developing countries (Kuschminder et al., 2012).

Minaye and Meseret (2016) stated that South Wollo Administrative Zone in Amhara Region is one of the zones where a high number of potential migrants are found. The *woredas* in the South Wollo zone are a good example of areas with a large number of migrants. In South Wollo Region, Wugdi, Kelela, Mekaneselam, Legabo, and Mekdela are among the *woredas* where there is a high prevalence of migrants to the Middle East with an increasing number every year. It was indicated that 3,063 (*M* = 1,484, *F* = 1,579), 2,133 (*M* = 1,297, *F* = 836), and 483 (*M* = 112, *F* = 371) returnees were registered at Jama, Kelela, and Mekedela *woredas*, respectively, in 2015 only (Seifemichael and Tarekegne, 2015).

Schools are the most potent sources of migration in the Amhara region in general and in the South Wollo Zone in particular. Migrant recruiters and peer influence to migrate are prominent at schools in the study areas. Students from grade 7 up to grade 12 are highly susceptible to migration. Students in these grade levels either migrated or have a plan to migrate for different reasons (Zelek, 2011; Yilma, 2013). Schewel (2018) also indicated that female students who failed an exam at the end of each cycle such as grades 8, 10, and 12 are a group who are at the most risk for migration. Since they lack independent life skills, as they have mostly lived with their parents, this early-aged migration might cause psychological and health problems for the migrants. This migration option might affect the opportunities of these students to further their education at a TEVT and their promotion to the next education levels and that in turn affects the cultivation of educated manpower in Ethiopia. This condition worsens the problem as most Ethiopian youngsters are considering education as a means to buy time for future migration.

Migrants spend a huge amount of money to process their migration. According to the study area's society's anecdotal narrations and testimony of some migrant returnees, migrants spend up to 50,000 birrs (close to one thousand USD) to process their migration. If this money is properly managed and invested, it could have enabled them to start their businesses in their own country where they live with their close families, relatives, and friends. Therefore, their intention to migrate and their behavior of migration seem to be beyond economic issues. This condition requires further research to investigate the social-psychological determinants of migration and migration intention in Ethiopia in addition to the economic factor which is over-examined.

In general, since the nature of migration is complex, it is unlikely that limited theories and disciplines can address all the issues it contains. It can be better understood if there is a synergy of a variety of disciplines or interdisciplinary studies concerning the nature of migration to deal with a range of variables associated with it. It appears that only the economic factor has been addressed as a determinant of migration and migration intention. However, focusing on economic factors might result in poor management of migration and migration intention. Therefore, it is time for psychology in general and social psychology, in particular, to come up with possible explanations concerning the nature and determinants of migration in Ethiopia.

Therefore, this study is designed to answer the following basic research questions:

- Do subjective social norms, attitude, national pride, place attachment, and perceived behavioral control predict the intention of high school students in South Wollo to migrate abroad?
- Does one's social network moderate the relationship between high school students' intention to migrate abroad and subjective social norms, attitude, national pride, place attachment, and perceived behavioral control?

### Objective of the study

The specific objective of this study was intended to:

- Identify if perceived behavioral control, subjective norms, national pride, place attachment, and attitude predict cross-border migration intention of high school students;
- Examine whether one's social network moderates the relationship between high school students' intention to migrate abroad and subjective social norms, attitude, national pride, place attachment, and perceived behavioral control.

### Theoretical framework

To accomplish this research, the theory of planned behavior is used as a theoretical framework. Several investigators showed the application of the theory of planned behavior in their attempts to predict and understand people's intentions to engage in various activities. The theory of planned behavior is very important to incorporate both individual and social factors in determining individuals' behavior, and it has been promoted by different researchers in studying the interaction effect of the social and psychological aspects on individuals' behavior. The theory can accommodate different theoretical constructs by including individual and social factors into the same model and has the capacity to predict a range of behaviors and intentions in numerous domains (Armitage and Conner, 1999; Ursin, 2016).

The theory of planned behavior is widely applicable to a variety of human behaviors to explain and identify predictors of individual behavior in diverse areas including health, environmental concern, mass transit use, risk communication, and technological adoption (Knabe, 2012). Ajzen (1991) also indicated that the theory of planned behavior is essential to draw an important conceptual framework to deal with complex human social behaviors. He stated that “The theory incorporates some of the central concepts in the social and behavioral sciences, and it defines these concepts in a way that permits prediction and understanding of particular behaviors in specified contexts” (p. 206). The theory of planned behavior has been applied to explain a variety of human behaviors including gambling behavior and the use of hormone replacement (Cameron, 2010). Similarly, in the review of the application of planned behavior, it was stated that this theory has been the most frequently cited and influential model applied in different research to explain human social behavior (Ajzen, 2011). In addition, Armitage and Conner (1999) indicated the role of the theory of planned behavior in explaining a variety of human behavior. They stated that the ability of the theory to predict a range of human behavior has been confirmed by different meta-analytic reviews of research on a variety of behavior by different researchers.

The important concepts that are considered in the theory of planned behavior to predict intention are attitude, subjective norm, and perceived behavioral control (Salehudin and Mukhlish, 2008). Intention is an essential immediate determinant of behavior and behavioral intention is determined by individuals' attitudes, subjective norms, and perceived behavioral control. “Intentions are indications of how hard people are willing to try, of how much of an effort they are planning to exert, to perform a behavior” (Ajzen, 1991). Attitude measures the degree to which the person has a positive or negative evaluation of the behavior to be performed. On the other hand, subjective norms refer to the belief of individuals of how significant others think about the appropriateness of performing that behavior. It is a social pressure exerted on an individual resulting from their perception of what other people around them think they should do and their tendency to comply with this norm. The perceived opinion of these significant others determines an individual's decision to act on the behavior. On the other hand, perceived behavioral control refers to the perception of individuals about their capacity to perform the behavior under consideration. As a general rule, Ajzen (1991) indicated that the more positive attitude toward the behavior, the more favorable the subjective norms with respect to that behavior, and the higher the perception of behavioral control to perform it, the stronger it would be for individuals to develop an intention to activate the behavior under consideration. To say it differently, an individual's behavior is best predicted by their intention whereby intention itself is determined by the attitudes of individuals toward the behavior, subjective norms are the person's perception about what significant others believe about the behavior, and perceived behavioral control, which is their perception about the capability they have to perform the action.

**Figure 1:**
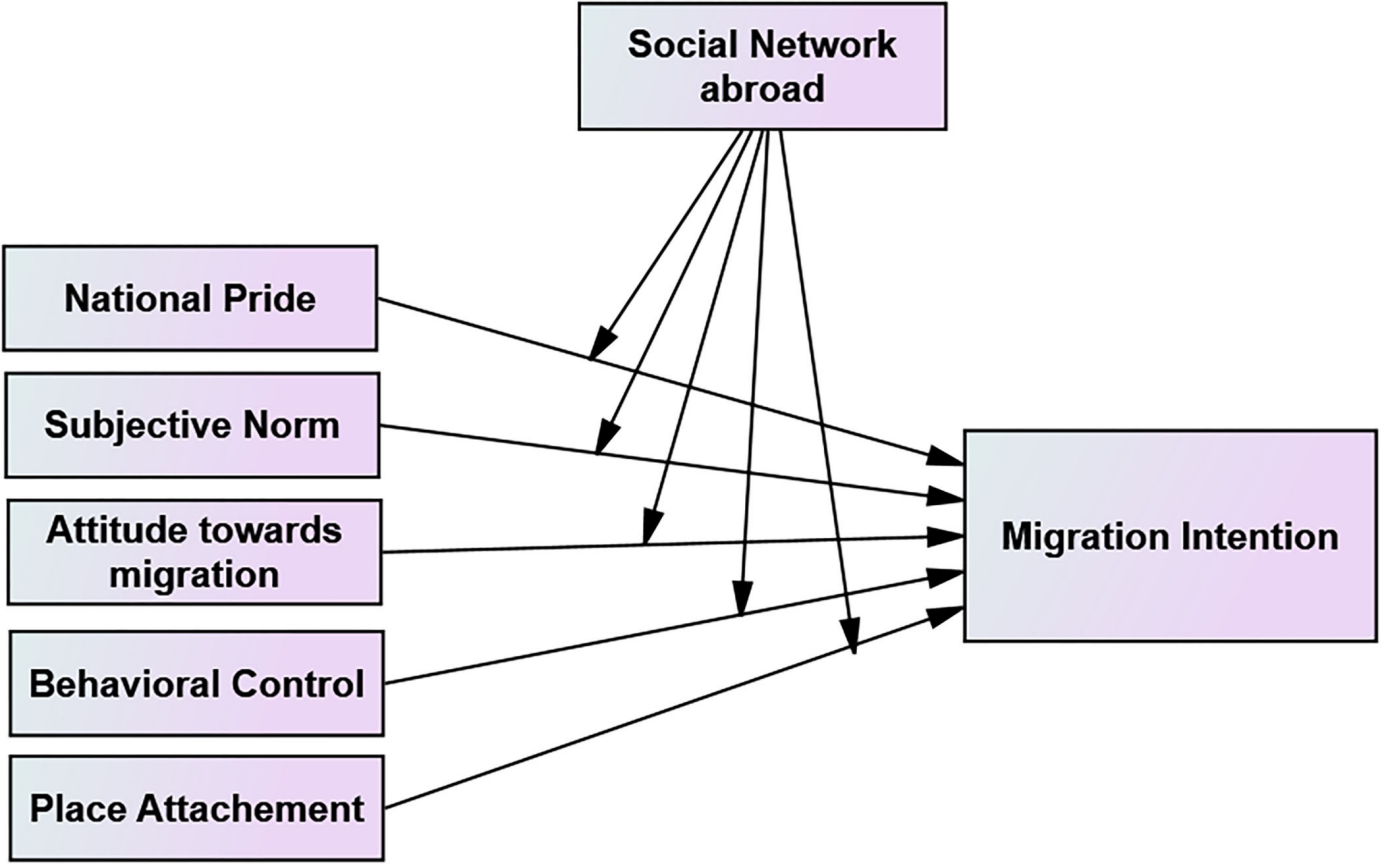
Conceptual frameworks to test the relationship and moderation effect of the variables.

The theory of planned behavior explains that individual behavior is based on conscious reasoned action and determined by a person's cognitive thinking and social pressures (Bhattacherjee, 2012). Behaviors are determined by intentions which, in turn, are predicted by attitudes, subjective norms, and perceived behavioral control. As one dimension of human behavior, the theory of planned behavior is applicable to the study of migration and migration intention. As a result of the importance of intentions, norms, networks, and constraints to study migration, the theory of planned behavior provides an important theoretical framework. In general, a review of different research findings showed that the theory of planned behavior is applicable to predict a range of behaviors from the intention the person holds. The review of research findings indicates that it should be clear that there are other variables (attitude, subjective norms, and behavioral control) that determine intention itself. More importantly, attitude, subjective norms, and behavioral control are not the only concepts included in the theory of planned behavior framework to explain human behavior. Other concepts and variables including background variables play a paramount role in determining intention. Above all, Ajzen (1991) pointed out that the relative importance of the three concepts (attitude, subjective norms, and behavioral control) in predicting intention might vary across behaviors and situations. “In some applications, it may be found that only attitudes have a significant impact on intentions, in others that attitudes and perceived behavioral control are sufficient to account for intentions, and in still others that all three predictors make independent contributions” (p. 188–189). As a result of these, in this particular research, an attempt was made to use the theory of planned behavior as the framework to investigate how the predictor variables determine the migration intention of individuals. The Figure 1 below shows the conceptual framework deemed to represent the relations among the predictor, dependent, and moderator variables.

## Research methods

### Research design

The research design that was employed in this research is an explanatory correlational design. This design is preferred to other designs as it indicates the relation of high school students' intention to migrate abroad, attitude, perceived behavioral control, subjective social norms, national pride, and place attachment. It also helps identify to what extent the selected social psychological factors predict the intention of high school students to migrate abroad. In support of these ideas, Creswell stated that explanatory correlational deign is essential to explain the association between two or more variable and researchers collects data at one point in time (Creswell, 2012).

### Population of the study

This study focuses on the western *woredas* of South Wollo as the area has a high potential for migration and there has not been extensive research as the *woredas* are too remote. The target population of this study was high school students in Borena, Jama, Kelala, Legambo, Legehida, Mehal Saint, Mekdela, Saint, Tenta, Wugidi, and Wore'elu *woredas*.

### Samples and sampling techniques

The South Wollo Administrative Zone was selected purposely as it is one of the potential areas of migration in Amhara Regional State. Data were collected twice. One was for item validation and the other was for the final study.

To select participants of the research for item validation, a multistage cluster sampling technique was employed. First, *woredas* from the western part of South Wollo were selected using simple random sampling (lottery method). At the onset, the names of the *woredas* were written on the paper. Then the pieces of paper were rolled and put in the hat. A student at Wollo University drew two pieces of paper with the names of the Mekdela and Tenta *woredas* on them. Once the *woredas* were selected, similar techniques applied to select *woredas* were applied to select the schools to draw the sample sections. With this sampling technique, Adjibar and Mekdela High Schools were selected.

Once the schools were selected using a simple random sampling technique (lottery method), information about the average number of students per class was collected from the principals of the schools. Since there was item validation, these gave a clue about the number of sections that would be included in the study as the number of participants for instrument validation should be >300 (Tabachnick and Fidell, 2013) students. The total number of students in Mekdela High School was 55 of which 26 of them were in grade 9, 10 of them were in grade 10, 11, and 8 were in grades 11 and 12, respectively. The average number of students per class in this high school was 41. The total number of students in Adjibar High School was 52. Out of the total students, 24 of them were in grade 9, 15 of them were in grade 10 and the other 8 and 5 were in grades 11 and 12, respectively. The average class size in Adjibar High School was 54.

Once the number of students in each grade level and the average number of students per class was known, the required section for the study was taken from all grade levels. A simple random sampling technique was employed again to choose sections in the school. Names of each section at different grade levels (9–12) were written on the pieces of paper and rolled. Teachers at these schools drew the sections that would participate in the study.

Four sections were taken from each school. The approximate total samples that ought to be taken from Mekdela High School were sample sections times average students per class (4^*^41) which were 164 and similarly, sample students from Adjibar (4^*^54) were 216. But the exact number of participants that were taken from the two schools was 165 and 214 from Mekdela and Adjibar High Schools, respectively. Therefore, the total number of participants in this research study was 379.

A similar sampling technique was used for the final study. Debrezeyit, Akesta, and Gimba High Schools were selected to participate in this final study. A total of 747 students from grade 9 to grade 12 participated. But out of the total participants, 701 students properly filled and returned the questionnaire.

### Instrument for data collection

Since there were no standardized instruments developed to measure the variables under study in Ethiopia, different instruments were adapted and validated to identify the latent variables through their indicators or observed variables. Respondents were requested to give their answers for each item on a 5-point Likert scale type from 0 (no one) to 4 (there are very much) for social networks and 1 (strongly disagree) to 5 (strongly agree) for intention, place attachment, subjective social norms, attitude, national pride, and perceived behavioral control.

The intention of individuals to migrate abroad was measured using the intention scale developed by Chan-Hoong and Soon (2011) and had five items with a Cronbach's alpha of 0.77. The scale was designed with a 5-point scale from 1 (strongly disagree) to 5 (strongly agree). A higher score denotes the individual's desire to migrate abroad. But the instrument does not have a determined cut-off point.

The attitude of individuals toward migration was measured using the scales developed by previous researchers. The attitude measuring instrument has social status and socio-economic security items in it. The social status having two items has a Cronbach's alpha of 0.6 and the socioeconomic scale with ten items has a reliability of 0.79 (Chan-Hoong and Soon, 2011).

Place attachment of individuals was measured with the adapted scale developed by Frieze et al. (2011) and had 30 items with a Cronbach's alpha of 0.93. These researchers conducted a factor analysis and the result indicated one factor with an eigenvalue of 10.08 accounting for 33.6% of the variance.

In order to measure the subjective social norm, items developed by Speelman et al. (2016) with a Cronbach's alpha of 0.77 and Chan-Hoong and Soon (2011) with a Cronbach's alpha of 0.53, with three questions each, were used. A total of six items (i.e., three from each) were used to measure this construct. A higher score indicates that the respondents are experiencing higher pressure from important persons to migrate.

Perceived behavioral control of individuals was measured using scales developed by Speelman et al. (2016) with a Cronbach's alpha of 0.74 and five questions. Respondents rated on a 5-point Likert scale how much they disagree or agree with each item about their ability to migrate abroad. The score ranged from 1-strongly disagree to 5-strongly agree with a higher score indicating a high ability to migrate. To measure the social network of the participants of this research abroad, the scale was adapted from the work of Lewicka (2011) with a reliability of 0.82 and 0.80 for bridging and bonding social capital. The original scale had 12 items. But the questionnaire was revalidated and reduced to nine items with a reliability of 0.67 and 0.76 for instrumental and emotional social capital, respectively. In the current research, eight items of the scale were used by dropping one item during the panel discussion as it was the repetition of another item (Toruńczyk-Ruiz and Brunarska, 2018).

Finally, the national pride of the participants was measured using the general national pride scale containing five questions. The original scale was adapted from Smith and Jarkko (1998) with various reliability scores across cultures, from 0.33 to 0.70 in the Philippines and Germany respectively in the original version, and validated by Chan-Hoong and Soon (2011), who found a reliability score of 0.46. The scale consists of items that investigate patriotism, national superiority, and allegiance. A higher rating score indicates a greater level of national pride with a score of 5 for participants who give extreme anti-national responses to each item and 25 for those who give only pro-national sentiments.

### Data analysis techniques

To test the moderation effect of social networks on migration intention and the predictor variables (attitude, national pride, place attachment, subjective norm, and perceived behavioral control), structural equation modeling was employed using Amos version 26.

### Reliability and validity of instruments

As the initial versions of the instruments were in English, the first step was to translate them into the Amharic language. Translation to Amharic was done by a language expert and psychologist. The language expert was an assistant professor in the Amharic language at Wollo University. In addition, to evaluate the translation from the perspective of psychological essence, an assistant professor in developmental psychology from the psychology department participated. Subsequently, backward translation from Amharic to English was made by other language experts and psychologists. This was made to verify if the translated items are similar to the original ones. To check the content validity of the instrument, a panel discussion was conducted. Four teachers (two assistant professors and two lecturers) from the psychology department at Wollo University were the panelists to evaluate if the instruments contained relevant content concerning the issues under investigation. The panelists evaluated if the measuring instruments have adequate coverage of the topics under investigation. Moreover, judgment was given about the cultural relevance and translation of the instruments.

#### Exploratory factor analysis

Exploratory factor analysis (EFA) was used to uncover dimensions of the underlying data set or examine which of the items have the strongest association with the latent variable (DiStefano et al., 2009). To investigate the dimension of the latent variables and identify the association of each item to the factors, exploratory factor analysis was used, using SPSS version 26.0. In the present research, oblique rotation was used to simplify and clarify the data structure. Oblique rotation is preferred to orthogonal rotation as it provides a realistic representation of how factors are interrelated. It is stated that if factors are uncorrelated, oblique rotation produces almost the same result that would be produced by orthogonal rotation (Brown, 2006). Moreover, there has been a flawed conventional argument that researchers need to use orthogonal rotation but in social science, correlation is expected among factors. Therefore, oblique rotation is preferred as it can produce nearly identical results with orthogonal rotation if factors are not correlated (Castillo et al., 2016). Similarly, Field indicated that orthogonal rotation is not meaningful for any data involving human beings as there is no independent psychological construct that is not related to other psychological constructs (Field, 2013). He also stated that there are arguments that indicate orthogonal rotation should not be used. A type of Oblique rotation method called Promax was used as it is fast and appropriate for large data sets (Field, 2013). Promax also begins with varimax orthogonal rotation (Thompson, 2004; Brown, 2006) and if the orthogonal rotation correctly identifies the factors, Promax provides an efficient route to the oblique solution (Loehlin, 2004).

The Kaiser–Meyer–Olkin test score (KMO), which is 0.95, confirmed the adequacy of the sample and Barlett's Test of Sphericity was significant (*p* < 0.01) and supported the factorability of the items. Using the Maximum Likelihood (ML) extraction method, the instruments of the seven variables (intention, subjective social norms, attitude, perceived behavioral control, national pride, place attachment, and social network) had 71 total items that were subjected to SPSS version 23. The analysis revealed the presence of seven components with eigenvalues exceeding 1, explaining 28.10, 12.94, 7.14, 4.6, 3.45, 2.79, and 1.87% of the variance, respectively. The seven components solution explained a total of 61% of the variance. In addition, factor analysis was also conducted for each scale to investigate if there are dimensions for each latent variable independently. The results indicated that there were no dimensions for all of the scales of the latent variable used in this study. The reliability of tools is indicated in Table 1.

**Table 1:** Reliabilities of instruments.

**Variable**	**Reliability**
Intention	0.91
Attitude	0.89
Perceived behavioral control	0.94
Subjective social norms	0.89
Place attachment	0.95
National pride	0.82
Social network	0.80

#### Confirmatory factor analysis

To validate to what extent the indicator variables are predicted by the latent variables, factor analysis was conducted using Amos version 26. One of the most commonly used factor analyses, confirmatory factor analysis, was employed to determine if the observed variables measured the factors under consideration.

Pooled confirmatory factor analysis was preferred to individual confirmatory factor analysis as it is easy and better able to determine the validity of the measuring items in relation to the latent constructs (Chong et al., 2014).

The ratio of chi-square to degrees of freedom was (CMIN/DF) = 1.071, the Comparative Fit Index (CFI) = 0.99, the Standardized Root Mean Square Residual (SRMR) = 0.038, the Root Mean Square Error of Approximation (RMSEA) = 0.014, and P-CLOSE = 1. Moreover, all the loadings of the items were significant and above 0.5, which indicated that more than 50% of the variance is explained by latent factors. Table 2 shows both the result of the confirmatory factor analysis and the acceptable ranges a nd their interpretation based on Gaskin and Lim (2016) cutoff criteria.

**Table 2:** Model fit measures of latent constructs.

**Measure**	**Estimate**	**Threshold**	**Interpretation**
CMIN	1451.993	–	–
DF	1356	–	–
CMIN/DF	1.071	Between 1 and 3	The requirement was achieved
CFI	0.991	>0.95	The requirement was achieved
SRMR	0.038	< 0.08	The requirement was achieved
RMSEA	0.014	< 0.06	The requirement was achieved
PClOSE	1.000	>0.05	The requirement was achieved

### Ethical considerations

Concerning the ethics of the research, the participants were told that they have the right to quit at any time or not to participate if they were not interested in it. Moreover, they were told that their information about the issue under study would be kept confidential. There were no personal identifiers used, just codes assigned for each questionnaire. In general, the research was conducted by taking the social and psychological well-being of the participants into account.

## Results and discussion of the study

### Result of the study

In this section, the collected data are presented and analyzed. The results began by describing the demographic characteristics of the participants of the study. Next to this, path analyses of variances were presented in line with the objectives of the study.

#### Demographic characteristics of participants of the study

A total of 747 high school students from different grade levels participated in the current research. The return rate of the questionnaire was 93.84% as 701 students filled correctly and returned it. Forty-six students, i.e., 6.16% of the total participants, either did not properly fill out or did not return the questionnaire and were not included in the analysis. The frequency and percentage of the sample in terms of school grade level and sex who responded correctly are presented in Table 3.

**Table 3:** Demographic characteristics of the sample.

**Demographic characteristics**		**Number (percentage)**
Sex	Male	394 (56.21%)
	Female	307 (43.79%)
	Total	701 (100%)
School grade level	9	255 (36.4 %)
	10	179 (25.5%)
	11	148 (21.1%)
	12	119 (17%)

#### Prediction and moderation effect on migration intention

**Figure 2:**
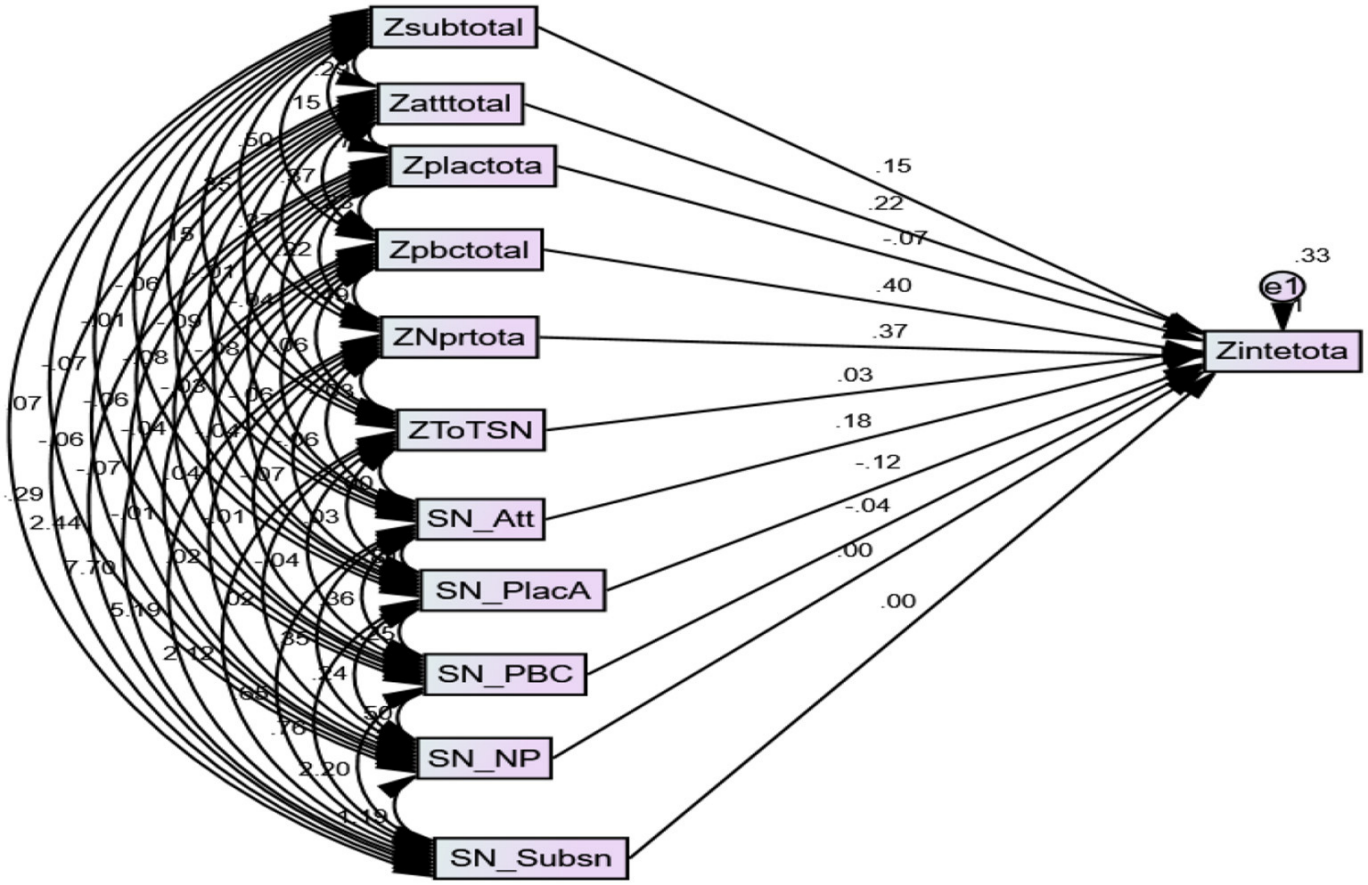
Path coefficients predicting migration intention from the latent variables and their interaction with the social network.

As it is portrayed in Figure 2, perceived behavioral control, national pride, attitude, and subjective social norms predicted an intention to migrate abroad significantly and positively. Perceived behavioral control was the strongest (β = 0.40, *p* < 0.001) predictor of an intention to migrate abroad of all other predictors. The result also indicated that national pride is the second strongest predictor (β = 0.37, *p* < 0.001) compared to the rest of the other independent variables. Attitude (β = 0.22, *p* < 0.001), and subjective social norms (β = 0.15, *p* = 0.040) also predicted an intention to migrate abroad. As was presented in the result, place attachment did not significantly (β = −0.066, *p* = 0.054) predict an intention to migrate abroad.

To examine the interaction (moderation) effect of social networks on attitude, place attachment, subjective social norms, perceived behavioral control, and national pride on an intention to migrate abroad, a path analysis was conducted.

Figure 2 shows the result of the analysis of both the interaction of social networks with other predictor variables and the coefficient of the predictor variables (attitude, place attachment, subjective social norms, perceived behavioral control, and national pride) and a criterion variable (intention to migrate). As it is portrayed in the Figure, the first step that was done before the analysis of the interaction effect of social networks and other independent variables on intention, the total score of each variable was standardized. Therefore, Zatttotal, Zplactotal, Zsubtotal, Zpbctotal, Ztotsn, Zintetotal, and ZNprtotal represent the standardized score of total scores of the variables in the research.

**Figure 3:**
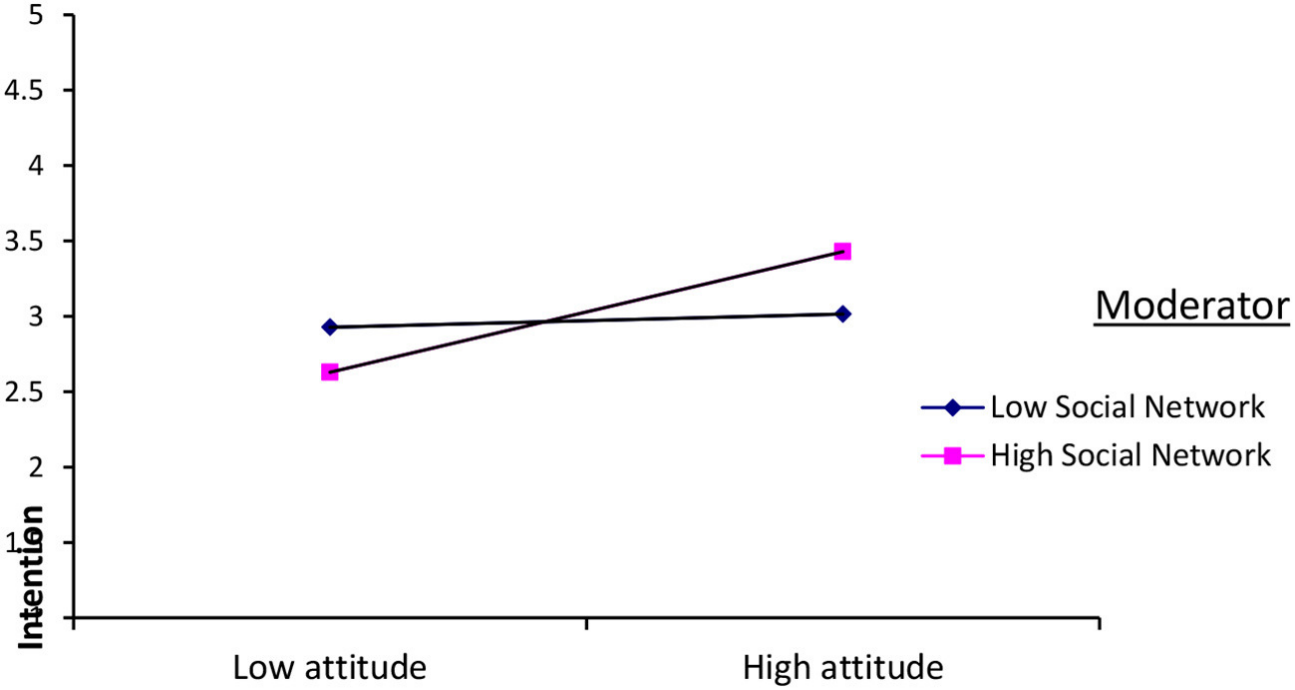
The plot of the interaction effect of attitude and social networks on intention to migrate.

As it is portrayed in Figure 2, social networks moderated the relationship between place attachment and migration intention, and attitude and migration intention. The result indicated that social networks positively (β = 0.18, *p* < 0.01) strengthened the relationship between attitude and an intention to migrate. However, the mere presence of social networks did not play role in strengthening the relationship between migration intention and attitude. It was indicated that low social networks did not have a moderation effect between the variables. But the relationship between attitude and migration intention was strengthened only when there was a high social network. Figure 3 below revealed how the nature of social networks strengthens the relationship between attitude and an intention to migrate abroad.

**Figure 4:**
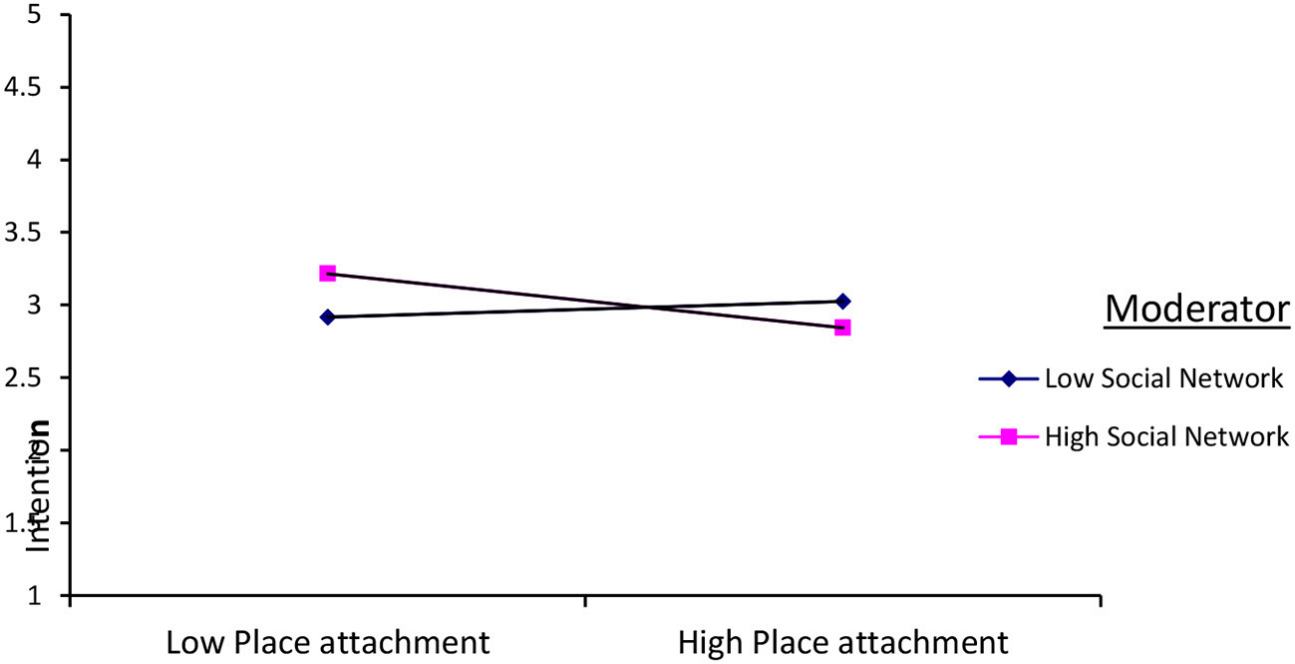
The plot of the interaction effect of place attachment and social network on intention to migrate.

The result of path analysis also indicated that social networks significantly (β = −0.12, *P* < 0.01) strengthened the negative relationship between place attachment and an intention to migrate abroad. But the moderation effect of social networks between place attachment and an intention to migrate was significant when the individuals' social network was high. However, when the social network was low, the interaction effect of social networks between the predictor and criterion variable was not significant. Figure 4 below shows the figurative representation of the interaction effect of social networks between place attachment and migration intention.

## Discussion

### Relationship between migration intention with attitude, place attachment, subjective social norm, perceived behavioral control, and national pride

Attitude is one of the determinants of behavior through intention. The result showed that attitude toward migration significantly predicts the intention to migrate abroad. This research finding was similar to other previous findings. Minaye and Zeleke (2017) stated that attitude is a good predictor of migration intention. Similarly, Ommundsen et al. (2010) indicated that as migration is perceived to be means of improving one's own life and family life, many young individuals develop a strong migration intention. In the same way, Chuvashov (2014) reported that attitude plays a great role in determining migration intention. Individuals with a positive attitude toward migration have a high degree of migration intention to other countries.

Attitude was a significant predictor of intention to migrate abroad for different reasons. Firstly, individuals who graduated from different levels of education institutions have not been employed. In every society, the unemployment rate seems to be increasing from year to year. This unemployment rate could affect vicariously the perception of the surrounding adolescents about their future fate. This in turn might lead to migration as one possible choice of job “opportunity” which, again, leads to the development of intention to migrate abroad among adolescents.

Another predictor variable that was investigated as a predictor of an intention to migrate was place attachment. The result of the present research indicated that place attachment did not significantly predict the intention of high school students to migrate abroad. The result of this study was similar to the finding of Eacott and Sonn (2006) who stated that place attachment does not significantly predict the migration intention of individuals. They found out that the attachment individuals have to a place did not significantly predict individuals' migration intention. But the result was contrary to other findings. For example, Frieze et al. (2011) stated that place attachment predicted individuals' migration intention. These researchers found that individuals who have a strong place attachment to their home country or residence areas together with having large friends increase the possibility of their stay in their place of origin. On the other hand, individuals who lack strong place attachment and have few friends in their place of origin are highly susceptible to migration and migration intention. It is also stated that individuals with a lower place attachment have a positive attitude toward migration (Petrović et al., 2017) and those with high place attachment develop the intention to stay where they are living (Toruńczyk-Ruiz and Brunarska, 2018).

As presented above, place attachment did not significantly predict the migration intention of adolescents. This could be explained in different ways. For example, participants of this study might not prioritize the emotional and physical attachment to the place they have lived but rather economic issues, the influence of the society, and perceived job opportunities could be dominant to affect their thinking about migration and intention to migrate. Individuals with high place attachment might migrate to take advantage of economic and education opportunities (Barcus and Brunn, 2009).

With regard to the relation between subjective social norms and an intention to migrate, the results indicated that subjective social norms significantly (β = 0.15, *p* 0.04) predicted the migration intention of high school students. This finding is similar to the findings of other researchers. For example, Gödri and Feleky (2017) revealed that subjective social norms determine individuals' emigration intentions. It predicts the migration behavior of individuals through migration intention. Similarly, DeJong (2000) indicated that the out-migration and migration intention of males and females is positively predicted by subjective social norms. The present research finding is also similar to the findings of McHugh (1984) which shows subjective social norms positively predict the migration intention of individuals. According to Willekens, components of the theory of planned behavior such as attitude, subjective social norms, and perceived behavioral control predict individuals' migration intention (Willekens, 2021). But the current research finding contradicted (Chuvashov, 2014) finding which indicated subjective social norms did not predict individuals' intention to migrate.

Subjective social norms' prediction of intention to migrate can be explained by the implicit and explicit influence of society in pushing adolescents to migrate. This might be due to the perception of society that their daughters and sons can be one source of income by migrating and working abroad. Particularly, when previously migrated individuals' parents' life is “changed”, because of the remittance, other members of the society consider it as a good opportunity to bring about life change for the migrants and their families which, in turn, shape the perception of adolescents about migration. This societal and family perception about migration might affect their behaviors which are manifested knowingly or unknowingly to influence the adolescents' or students' migration intention.

This research finding also revealed that perceived behavioral control significantly predicts the migration intention of adolescents. The finding of this research was similar to the findings of Groenewold et al. (2012) who stated that perceived behavior control predicted migration intention. McHugh (1984) also found out that perceived behavior control was one of the social-psychological determinants of migration intention. But the finding of the current research contradicts the findings of Chuvashov (2014) who stated that perceived behavioral control did not predict individuals' intention to migrate.

Researchers stated that the perception of individuals about their capability to perform an activity determines their development of intention which is the immediate predictor of behavior. Individuals' evaluation of their capacity to overcome an activity is essential to develop the intention to engage in that activity (Ajzen, 1991; Salehudin and Mukhlish, 2008). In the present research, it was identified that perceived behavioral control significantly and positively predicted migration intention. This could be explained by a variety of views. One of the most important challenges to migrating is getting enough money to accomplish the migration. In this regard, adolescents might not think they will face such a problem as society is assumed to have a positive attitude toward migration. Adolescents might perceive they will access enough money to accomplish their migration from society which in turn influences them to develop the intention to migrate abroad. In addition, it has been common to have individuals who migrated from nearby villages, kebeles, and *woredas*. These previously migrated community members play a paramount role in facilitating the migration process including finding jobs, giving temporary accommodation (Massey et al., 1993), and simplifying the challenges the newly arrived migrants face by providing support (Ryan et al., 2008). This results in easing migration and leads to developing migration intention abroad. Moreover, the adolescents might have not well accessed the negative impact of migration both on the way, at the terminals, and destinations. Above all, as adolescence is the age of transition at which adolescents try to separate from their parents, they might take migration as an opportunity to experience a new life and decide their future.

As the results of this research indicated, national pride significantly predicted migration intention. This research finding was similar to other previous research findings in that national pride predicted an intention to migrate but in a different way. For example, Chuvashov (2014) found that national pride negatively predicted the migration intention of individuals. Chan-Hoong and Soon (2011) also stated that national pride and migration intention are negatively correlated. The findings of the above researchers indicated national pride negatively predicted the migration intention of individuals. However, the present research indicated that national pride positively predicted the intention to migrate abroad. This could be the manifestation of the role of cultural differences and immediate realities. The previous research findings which stated that national pride negatively correlated to migration were conducted in countries like Singapore where the economic conditions, recreational environment, education access, job opportunities, and technological advancement were better than in Ethiopia. Because of these reasons, individuals with a high sense of national pride are more likely to want to serve their nation. However, the current Ethiopian context is different and far from Singapore's reality. According to Smith and Jarkko (1998), national pride is related to one's feeling of patriotism and nationalism. Patriotism is the love of one's country or dedicated allegiance to the same, while nationalism is a strong national devotion that places one's own country above all others. If national pride is characterized in such a way, adolescents who were participants in this research might be committed and devoted to alleviating the present multidimensional problems of Ethiopia. They might think they could help their country, in general, and their society, in particular, by using migration as one instrument to achieve goals like economic development and technology transfer.

There are research findings that state migration is one of the means that contribute to the development of a nation. Migration is one means of poverty reduction that can positively change the life of migrants, their families, and the community as a whole (Foresti et al., 2018). In addition, OECD/ILO reported the acknowledgment of the 2015 Addis Ababa Agenda and the 2030 Agenda of Sustainable Development concerning the contribution of migration to the economic development of the sending, transit, and destination countries. It is indicated that the average contribution of migrants to the gross domestic product (GDP) can reach 7% (OECD/ILO, 2018). Migrants contribute to poverty reduction by increasing income through remittance, increasing the establishment of new businesses by providing start-up financial resources, and transferring new knowledge and skills for doing business (Ratha et al., 2011). It is indicated that, in addition to the contribution of economic development through remittance, migration is helpful in the reduction of the unemployment rate which is becoming one of the challenges for societies and governments around the world. Therefore, the reason why national pride positively predicted the intention to migrate abroad in the present research could be due to the commitment of the adolescents to help their country in its development by considering migration as one of the options for the development of a country.

#### The interaction effect of social networks on migration intention

With regard to the moderation effect of social networks in the relationship between the predictor variables (place attachment, attitude, subjective social norms, perceived behavioral control, and national pride) and the outcome variable (migration intention), the result indicated that the moderation effect of social network is revealed only in two predictors and migration intention. It moderated and strengthened the positive relationship between attitude and migration intention. But the moderation effect was significant when adolescents have a high social network. On the contrary, when the adolescents' social network was low, its moderation effect was not significant.

Among the different means, attitude is formed is through social learning. Social learning is one of the mechanisms to form an attitude. People learn a behavior and behavioral intention through vicarious learning and make cognitive inferences based on what they experience from others (Bandura, 1997). In line with this principle, when individuals have a high social network, it increases immediate and “imagined” social interaction and connection which in turn enhances their migration behavioral intention. Attitude remained potent when information about attitude objects is frequently accessed. The relationship between attitude and behavioral intention and behavior is strong when the attitude is accessible, and attitude is accessible when frequent information and related symbols concerning the attitude objects are available (Myers, 2010). When individuals have a high social network, they might get opportunities to contact them through different mechanisms, including social media. The remittance, the businesses opened by migrants and their families, and other technology-related materials make adolescents' attitudes potent and determine their migration intention. Moreover, the cognitive element of attitude is directly impacted by information coming from the external environment. The cognitive dimension of attitude is influenced by communication between or among individuals (Fishbein and Ajzen, 1977). As a result, when there is a high social network for adolescents, the exchange of information about migration will be high. This exchange of information about migration makes the individuals' attitude easily accessible and enhance their intention to migrate abroad.

The result of path analysis also indicated that social networks significantly strengthened the negative relationship between place attachment and migration intention. But the moderation effect of social networks between place attachment and migration intention was significant when the adolescents' social network was high. On the contrary, when the social network was low, the interaction effect of social networks between the predictor and the criterion variable was not significant. When adolescents have a low place attachment and have a high social network, their migration intention will be high. This could be justified because if they have a high social network, they might think that previously migrated individuals facilitate their migration process. The previously migrated members of the society serve as social capital for those who intend to migrate abroad in search of jobs, providing them with accommodation, giving information, and delivering psychological support. In addition to getting information about the existing jobs and conditions in the destination country, the social Network serves as a safety net by providing them with material and social support (Chi, 2020).

Massey et al. (1993) found that migration is predicted by the interpersonal ties which connect the potential migrants, the already migrated individuals who lived abroad, and non-migrants in the origin and destination countries through different linkages such as kinship, friendship, and shared community origin. This social capital minimized the cost of migration, maximized the benefits migrants could achieve from migration, and made it easy to find jobs in the destination countries. Similarly, (Zaiceva and Zimmermann, 2009) also stated that the existence of networks and a migration chain reduced the risks and costs of the migrants which, in turn, increases migration. The previous migrants provide accurate information about the reality like the available employment opportunities in the destination countries for the potential migrants.

## Conclusion and implication

As migration is one of the critical issues with far-reaching positive and negative impacts for Ethiopia, interdisciplinary research is required. In line with this view, this research was conducted to explain migration from a social-psychological perspective. Previous studies focused on the economic dimension. The present research focused on some social-psychological variables to identify if they predict migration intention. Accordingly, attitude, place attachment, perceived behavioral control, subjective social norms, and national pride were considered to investigate whether they determine the migration intention of adolescents. The results indicate that all the above variables, except place attachment, predict migration intention. Perceived behavioral control was found to be the top predictor. This is conceptually meaningful as self-efficacy is a core psychological variable that shapes intention and action. The fact that place attachment failed to predict migration intention can be linked to the overweighting power of unemployment and the consequent desperation and desire for change. In other words, the adolescents were becoming pragmatic and realistic in that remaining at one loved origin place without a job and hope does not make them productive.

Moreover, this research identified the moderation effect of social networks between the criterion variable (migration intention) and the predictor social psychological variables. The result indicated that social networks positively moderated the relationship between attitude and migration intention. On the other hand, social networks strengthened the negative relationship between place attachment and migration intention when individuals have a high social network. What is interesting here is that a social network's capability to moderate is significant when the social network is high. When the social network is high, the support or distraction that adolescents receive from the network is high, thereby having a profound positive or negative impact on their social behavior. Currently, we have not found a significant moderation effect of social networks between the three predictor variables (perceived behavioral control, national pride, and subjective social norms) and the criterion variable, migration intention, implying the need for further study in other settings.

Finally, the current research hints that it is not only economic issues that determine migration intention. Social psychological issues are one of the important determinants of migration intention. Therefore, more interdisciplinary research is required to better understand migration and migration intention to develop a policy for the management of migration. For example, an anthropological study on the impact of culture on migration intention would be useful.

## Data availability statement

The raw data supporting the conclusions of this article will be made available by the authors, without undue reservation.

## Ethics statement

The studies involving human participants were reviewed and approved by Ethical Committee of Psychology Department. Written informed consent to participate in this study was provided by the participants' legal guardian/next of kin.

## Author contributions

Both authors listed have made a substantial, direct, and intellectual contribution to the work and approved it for publication.

## Conflict of interest

The authors declare that the research was conducted in the absence of any commercial or financial relationships that could be construed as a potential conflict of interest.

## Publisher's note

All claims expressed in this article are solely those of the authors and do not necessarily represent those of their affiliated organizations, or those of the publisher, the editors and the reviewers. Any product that may be evaluated in this article, or claim that may be made by its manufacturer, is not guaranteed or endorsed by the publisher.
